# Antennae of psychodid and sphaerocerid flies respond to a high variety of floral scent compounds of deceptive *Arum maculatum* L.

**DOI:** 10.1038/s41598-022-08196-y

**Published:** 2022-03-24

**Authors:** Eva Gfrerer, Danae Laina, Rüdiger Wagner, Marc Gibernau, Anja C. Hörger, Hans Peter Comes, Stefan Dötterl

**Affiliations:** 1 Department of Environment and Biodiversity, Paris Lodron University of Salzburg, 5020 Salzburg, Austria; 2 Department of Limnology, University of Kassel, 34127 Kassel, Germany; 3 Laboratory of Sciences for the Environment, CNRS – University of Corsica, 20000 Ajaccio, France

**Keywords:** Ecology, Chemical ecology

## Abstract

Insect-pollinated plants often release complex mixtures of floral scents to attract their pollinators. Yet scent compounds eliciting physiological or behavioural responses in pollinators have only been identified in few plant species. The sapromyiophilous aroid *Arum maculatum* releases a highly diverse dung-like scent with overall more than 300 different compounds recorded so far to attract its psychodid and other fly pollinators. The volatiles’ role in pollinator attraction is mostly unknown. To identify potential behaviourally active compounds, we recorded electroantennographic responses of four Psychodidae and one Sphaeroceridae species to (1) inflorescence scents of *A. maculatum* and (2) the scents released by cow dung, likely imitated by the plant species. Here we show that these flies are sensitive to 78 floral volatiles of various chemical classes, 18 of which were also found in cow dung. Our study, which for the first time determined physiologically active compounds in the antennae of *Psychoda* spp. and Sphaeroceridae, identified various volatiles not known to be biologically active in any floral visitors so far. The obtained results help deciphering the chemical basis that enables *A. maculatum* and other plants, pollinated by psychodids and sphaerocerids, to attract and deceive their pollinators.

## Introduction

Floral scents are important mediators of plant–animal interactions^1^. Typically, a floral scent bouquet is composed of 20–60 volatiles per plant species^2^, but some species emit highly complex bouquets consisting of more than 100 volatiles^3–5^. In generalist pollination systems, widespread compounds (e.g., 2-phenylethanol, phenylacetaldehyde, benzaldehyde^6–8^) are typically involved in pollinator attractions, whereas in specialised systems^9^, the specificity in pollinator attraction is often reached by the emission of either specific blends composed of common compounds^10^ or highly specific compounds. Examples of such particular compounds are diacetin, which attracts highly specialised oil-collecting bees^11,12^, 4-methyl-5-vinylthiazole, which attracts cyclocephaline beetles^13–15^, or *p*-cresol as well as dimethyl trisulphide, which attract flies seeking oviposition sites^16,17^. Although the number of compounds known to be involved in pollinator attraction is increasing, the biological role of the more than 2,000 floral volatile organic compounds (VOCs) that have already been described^2,18^ is largely unknown.

In plant species with highly complex bouquets, it is often not possible to obtain all floral compounds as pure substances and to disentangle their individual behavioural effects. Additionally, many scent compounds are often not identifiable because characterised references are missing and are then listed as unknowns, which complicates investigations of their biological role^4,5^. In consequence, there are large gaps in our understanding of the chemical communication, especially between plants with complex scents and their pollinators.

**Figure 1:**
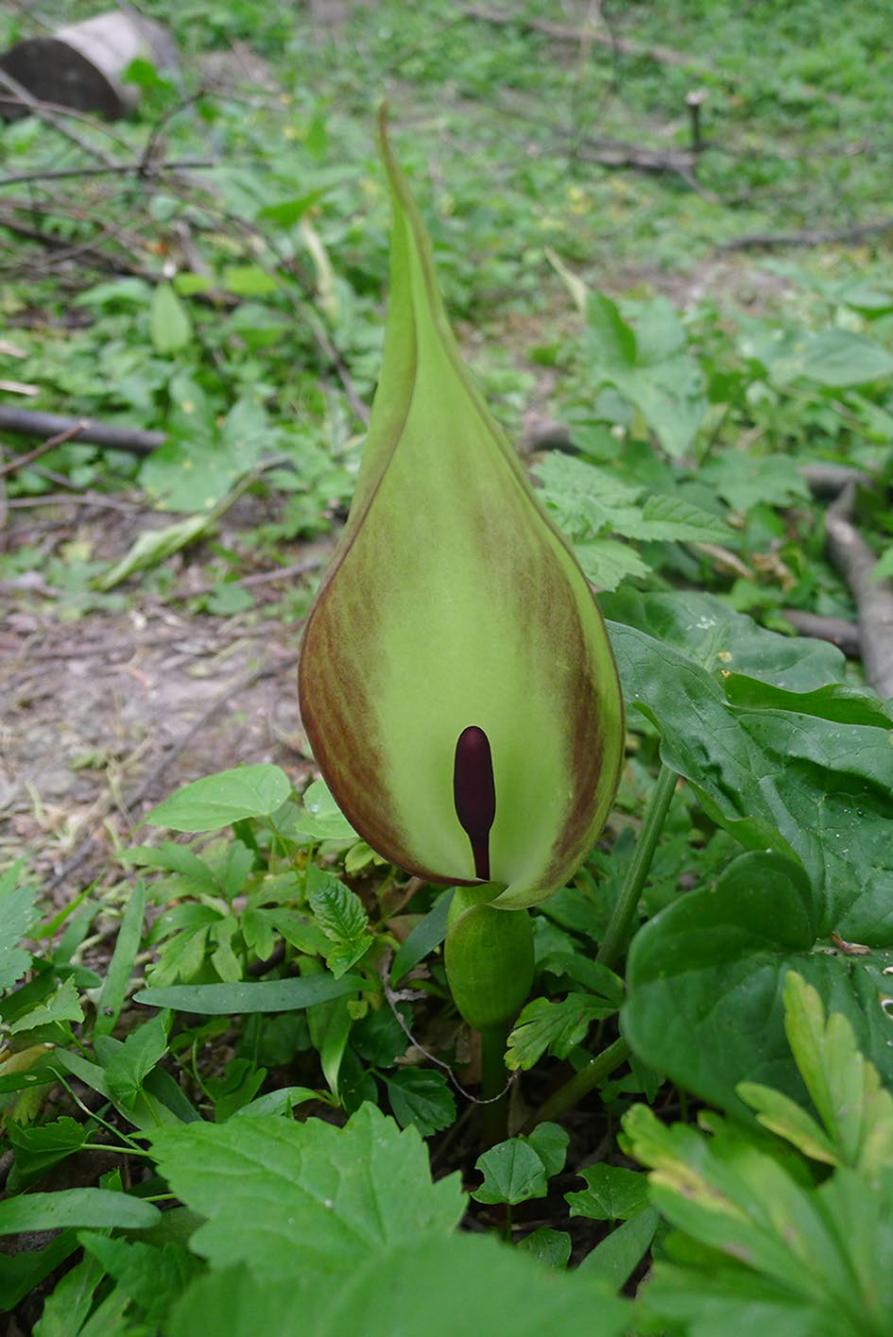
A flowering individual of *Arum maculatum* in its natural habitat. (Photo E. Gfrerer; Salzburg, Austria).

The fly-pollinated and brood-site deceptive *Arum maculatum* L. (Araceae, Fig. 1), a widespread European perennial herb, emits a remarkably diverse scent. A single individual releases up to 150 different scent compounds^5^, and overall more than 300 compounds have been recorded for this plant species so far^5,19–23^. However, most of these compounds have not yet been identified^5^. Among the identified compounds, some are widespread floral scents (*e.g.,* methyl salicylate, germacrene D) and/or well-known from brood-site deceptive plants (*e.g.,* indole, *p*-cresol, 1-octen-3-ol)^16^, while others are rarely found as floral scent compounds (*e.g., β*-lutidine, *p*-cresyl butyrate)^2,18^.

In Central and much of Western Europe, *A. maculatum* is predominantly visited and pollinated by female *Psychoda phalaenoides* L. (Psychodidae, Diptera)^24,25^. Yet, in Western France and the Mediterranean region, the most abundant visitors are often other psychodid species (*e.g*., *P. grisescens* tonn., *P. trinodulosa* tonn., *P. zetterstedti* ježek) as well as non-psychodid Diptera, such as Sphaeroceridae^24,26,27^. Psychodidae and Sphaeroceridae are rare pollinators of angiosperms, but apart from *A. maculatum* also pollinate other species of *Arum* (*e.g., A. concinnatum*, *A. cylindraceum, A. cyrenaicum, A. italicum,*) and other species/genera of Araceae (*e.g., Typhonium eliosurum*, *Arisaema heterocephalum*)^28–32^. In *A. maculatum,* insects are attracted on the first day of anthesis (female stage) to the pitfall-trap inflorescence (Fig. 1). They slip and fall into the floral chamber (i.e., the lower part of the inflorescence where the fertile flowers are situated), where they are trapped overnight and are only released on the second and final day of flowering, after being dusted with fresh pollen^19,32,33^.

The strong, dung-like inflorescence scent of *A. maculatum* attracts its fly-pollinators^21,34^ which breed in (and mate on) a range of different decaying organic matter, such as moist leaf litter, mushrooms, cow or horse dung^35–38^. Although the pollination biology of *A. maculatum* is well-studied^31–33^, the role of the individual floral volatiles for pollinator attraction remains largely unknown. To date, only five compounds of *A. maculatum* (*i.e.*, indole, *p*-cresol, 2-heptanone, *α*-humulene, and skatole)*,* all also released by cow dung^39,40^, have been tested for attractiveness to potential pollinators, and their attractive function in (female) Psychodidae has been proven^28,34,41^. However, preliminary bioassays at two natural sites of *A. maculatum* (Salzburg/Austria; Daone/North Italy) could not verify that these compounds, alone or as a blend, are attractive to psychodid (and sphaerocerid) flies, suggesting that other compounds are more likely to be responsible for the main attractiveness of the inflorescence (Gfrerer et al*.*, unpubl.).

Compounds potentially behaviourally active in plants with complex scents, such as *A. maculatum,* can be pre-selected by measuring the insects’ peripheral olfactory detection of floral scents, using gas chromatography coupled to electroantennographic detection (GC–EAD)^42,43^. In previous studies testing scent of various plant species (*e.g., Ceropegia sandersonii*^44^*, Encephalartos villosus*^45^), such measurements frequently pointed to candidate attractants and subsequently allowed the identification of behaviourally active compounds of (complex) scent blends^﻿44–46^. A comparison of the physiologically active floral compounds with the physiologically active compounds of the imitated substrate (or element) also helped to identify the key compounds of the plant–pollinator interactions^47,48^. Here, we investigated the antennal responses of four psychodid and one sphaerocerid species to the inflorescence scents of *A. maculatum* and to scent released by cow dung. Specifically, we asked (1) which of the many compounds recorded from this plant species elicit antennal responses in inflorescence visitors, (2) whether antennal responses differ among insect species, and between sexes within species, and (3) how many of the EAD-active scent compounds are shared between *A. maculatum* and cow dung. The study overall aimed to identify potential scent candidates responsible for attracting and deceiving different pollinators of *A. maculatum*.

## Material and methods

### Insect sampling

During 2017–2020, we obtained insects at two natural sites of *A. maculatum*, one located in Salzburg (Austria, 47°46′59"N 13°04′30"E) and the other in Marktschellenberg (Germany, 47°41′05"N 13°03′30"E). In 2017 and 2018, containers (5 L microboxes, Model: TP5000 + TPD5000–18.5 cm × 18.5 cm × 19.1 cm; Combiness nv, Nevele, Belgium) filled with fresh cow dung (c. 2 L) were offered to insects for three consecutive days as oviposition substrate at the Salzburg site (April–October). Afterwards, the containers were brought to the lab. Once psychodids and sphaerocerids started to hatch in the boxes, the flies were transferred to a small outdoor flight cage (60 cm × 60 cm × 60 cm; BugDorm, Talchung, Taiwan), in which they were offered different breeding substrates (soil, leaf litter, mushrooms, cow or horse dung). Insects reproduced in the cage and were available for electrophysiological measurements for four to six weeks, depending on the species. In spring 2019 and 2020, all insects used for measurements were obtained by bagging *A. maculatum* individuals with mesh bags at both sites in the morning of the second day of anthesis, prior to the release of trapped insect visitors. Once released by the plant and trapped in the bag, the flies were transferred to a flight cage and bred as described above.

### Floral scent collection, electrophysiological analyses (GC-EAD), and identification of EAD-active compounds

To acquire solvent scent samples for electroantennographic analyses, we collected inflorescence scent of *A. maculatum* and volatiles released by cow dung using dynamic headspace methods, following^44,49^. Plant volatile samples were collected from a total of eight populations (see Supplementary information Table S1), covering most of the observed scent diversity of *A. maculatum*^5^, whereas dung volatiles were obtained from cow dung samples (fresh or 1-day old) used for the rearing of flies (see above). Each inflorescence was enclosed in a plastic oven bag (c. 30 cm × 12 cm; Toppits, Melitta, Germany) on the first day of anthesis between 17:30 and 20:00, when scent emission is strongest^5,22^. Circa 60 mL dung was placed into 250 mL glass jars, covered with a plastic oven bag (see above). Volatiles were collected on adsorbent tubes (length: 8 cm, diameter: 2 mm), filled with a mixture of Tenax-TA (mesh 60–80) and Carbotrap B (mesh 20–40; 10 mg each; both Supelco, Germany), that were inserted through small holes into the headspace of the inflorescence and dung each. Samples were collected for 0.5–1.5 h with a flow of 100 mL min^−1^, generated by a battery-operated vacuum pump (rotary vane pump G12/01 EB, Gardner Denver Austria GmbH, Vienna, Austria). Due to the thermogenic activity of the inflorescence^22,50^, we partly opened the plastic oven bag at the top to avoid strong condensation of water inside the bag. Samples were eluted from each adsorbent tube using 80–100 µL acetone (SupraSolv, Merck KgaA, Germany; following^49,51^). Then, samples were pooled per population (plant) or per age (dung, *i.e*., fresh or 1-day-old) to be used for the physiological measurements. To confirm physiological responses to specific compounds, we recorded antennal responses to mixtures of synthetic compounds for a subset of compounds found in *A. maculatum* (Table 1, Supplementary information Table S1).

**Table 1:** Inflorescence scents of *Arum maculatum* that elicited electroantennographic responses in *Psychoda phalaenoides, P. zetterstedti, P. cinerea, P. trinodulosa, P.* sp*.* (Psychodidae) and *Coproica ferruginata* (Sphaeroceridae). Compounds are sorted according to chemical classes, and within those alphabetically. The volatiles printed in bold were also detected in cow dung. In parentheses, the numbers of individuals tested (n) and runs performed per fly species and sex are denoted. Superscript values indicate the amounts (ng per injected sample; mean±SD) of volatiles tested in the GC-EAD measurements on different fly species and sexes.

Species		*P. phalaenoides*		*P. zetterstedti*		*P. cinerea*	*P. trinodulosa*	*P.* sp.	*C. ferruginata*
Sex		♀	♂	♀	♂	♀	♂	♀	♀
#Individuals, #runs		(*n* = 12, 33)	(*n* = 8, 14)	(*n* = 2, 6)	(*n* = 2, 4)	(*n* = 2, 6)	(*n* = 1, 1)	(*n* = 2, 8)	(*n* = 1, 3)
Chemical class/compound									
*Aliphatic compounds*									
**Butanoic acid**^S^		+ + + + ^0.6±1.2^	*NP*	*NP*	*NP*	*NP*	*NP*	+ + + + ^0.7±1.6^	*NP*
**Decanal**^S^		+ + ^1.9±1.1^	+ ^1.3±0.9^	+ + + + ^0.5±0.8^	*NP*	+ + + ^1.0±1.4^	+ + + ^3.4^	+ + + + ^1.3±1.2^	+ + + + ^1.4±1.6^
**2-Decanone**		+ + + + ^0.4±0.5^	*NP*	+ + + + ^0.9±0.4^	*NP*	+ + + ^0.3±0.5^	*NP*	+ + + + ^0.3±0.3^	+ + + + ^0.4±0.7^
2-Heptanol*^S^		+ + ^5.8±10^	+ + + ^8.9±11^	+ + ^2.1±3.6^	+ + ^6.6±9.0^	+ + + ^20.5±27^	*NP*	+ + ^4.4±7.1^	+ + + ^18.7±27^
2-Heptanone*^S^		+ + ^14.8±17^	+ ^22.2±22^	+ + + ^24.5±27^	+ + + + ^17.6±18^	+ + + ^28.7±35^	*NP*	– ^15±13^	+ ^28.8±33^
1-Hexanol^S^		*NP*	*NP*	*NP*	*NP*	*NP*	*NP*	*NP*	+ + + ^0.2±0.2^
(*Z*)-3-Hexenyl acetate^S,L^		+ + + ^0.5±0.9^	*NP*	*NP*	*NP*	+ + ^0.5±1.1^	*NP*	*NP*	*NP*
**Nonanal**^S,L^		+ + ^1.3±1.6^	*NP*	+ + ^0.8±0.7^	*NP*	– ^0.6±0.9^	– ^2.6^	+ + ^0.5±1.0^	+ + ^0.3±0.5^
2-Nonanol^S^		+ + ^0.4±0.7^	*NP*	+ + + ^1.5±2.6^	*NP*	+ + ^3.5±4.7^	*NP*	+ ^0.3±0.5^	+ + + ^3.1±4.9^
2-Nonanone*^,S^		+ + ^2.1±1.6^	+ + ^3.0±2.7^	+ + + ^3.9±3.7^	+ + ^2.7±1.9^	+ + + ^5.4±6.7^	*NP*	+ + + ^1.9±1.7^	– ^5.16±6.5^
2-Octanone^S^		+ ^0.6±0.6^	*NP*	– ^0.5±0.3^	*NP*	*NP*	*NP*	+ ^0.5±0.7^	*NP*
3-Octanone^S^		+ + ^2.4±2.0^	*NP*	+ + + + ^0.3±0.4^	+ + + + ^4.0±1.6^	+ + + ^3.7±4.8^	*NP*	+ + ^3.1±4.4^	+ + + + ^5.7±5.5^
1-Octen-3-ol*^,S^		+ + + ^41.9±31^	+ + + ^32.9±18^	+ + + ^1.71±2.0^	+ + + ^54.1±29^	+ + + ^119±130^	+ + +^34.2^	+ + + ^27.5±28^	+ + + + ^111±132^
(*E*)-2-Octen-1-ol^S^		+ ^0.3±0.5^	*NP*	*NP*	+ + + + ^0.4±0.6^	+ + + ^4.0±8.4^	*NP*	*NP*	+ + + + ^6.7±10^
2-Undecene^S^		+ + ^5.2±15^	*NP*	*NP*	*NP*	*NP*	*NP*	*NP*	*NP*
*Aromatic compounds*									
*p*-Cresol*^,S^		+ + + ^56.4±45^	+ + + ^39.6±12^	+ + ^13.4±9.7^	+ + ^92.0±53^	+ + + ^41.9±49^	+ + + ^0.2^	+ + ^41.6±38^	+ + + + ^52.2±67^
Methyl salicylate^S^		+ + ^1.9±1.4^	+ ^1.1±1.1^	+ + + + ^2.1±1.2^	+ + + + ^2.2±1.9^	+ + + ^1.6±2.1^	+ + + ^0.8^	+ + + + ^1.3±1.7^	+ + + + ^1.4±2.4^
*Irregular terpene*									
6-Methylhept-5-ene-2-ol		+ + ^2.3±1.2^	*NP*	*NP*	*NP*	*NP*	*NP*	+ + + ^1.5±1.7^	*NP*
**6-Methylhept-5-ene-2-one**^S^		+ + ^2.8±1.4^	*NP*	– ^1.8±1.5^	+ + + + ^2.5±1.6^	– ^1.0±1.3^	*NP*	+ + ^1.9±2.2^	+ + + + ^1.3±1.4^
*Monoterpenoids*									
( +)-*α*-Citronellene*^,S^		+ + + ^9.2±6.2^	*NP*	+ + ^2.4±3.5^	– ^14.6±1.5^	+ + + ^6.9±5.1^	*NP*	+ + + ^6.3±4.2^	+ + ^9.6±3.9^
( +)-*β*-Citronellene*^,S^		+ + + ^93.8±69^	+ + + ^93.8±106^	+ + + ^28.7±31^	+ + + + ^160±55^	+ + ^105±91^	*NP*	+ + + ^90.4±55^	+ + + + ^142±50^
*β*-Citronellol^S^		+ + ^2.4±2.5^	*NP*	+ + ^0.6±0.9^	*NP*	+ + + ^4.6±5.9^	*NP*	+ + + + ^0.8±1.2^	*NP*
***p*-Cymene**^S^		– ^1.6 ±0.9^	*NP*	*NP*	*NP*	– ^0.3±0.7^	*NP*	*NP*	+ + ^0.9±0.6^
2,6-Dimethyloct-7-en-2-ol		+ + + ^4.8±2.9^	*NP*	+ + + + ^6.8±3.7^	*NP*	+ + + ^2.7±4.6^	*NP*	+ + + ^3.2±0.2^	+ + + + ^4.6±5.4^
2,6-Dimethyloct-7-en-4-one		+ + ^0.5±0.6^	*NP*	*NP*	*NP*	*NP*	*NP*	+ + + + ^0.5±0.3^	+ + + + ^0.3±0.3^
2,6-Dimethylocta-2,6-diene isomer 1*^,S^		+ + ^0.4±0.6^	+ + ^0.3±0.5^	– ^0.1±0.2^	+ + ^1.0±0.8^	+ + ^0.4±0.6^	*NP*	+ ^0.2±0.2^	– ^0.6±0.7^
2,6-Dimethylocta-2,6-diene isomer 2*^,S^		+ + + ^19.1±15^	+ + + ^14.5±2.2^	+ + + ^4.1±5.3^	+ + + + ^32.6±20^	+ + + ^17.9±9.1^	+ + + ^1.4^	+ + + + ^8.8±5.1^	+ + + + ^19.0±10^
2,7-Dimethylocta-4,6-dien-2-ol		*NP*	*NP*	+ + + + ^0.1±0.2^	+ + ^0.7±1.2^	*NP*	*NP*	*NP*	*NP*
3,7-Dimethyloct-1-ene*^,S^		+ + + ^30.8±27^	+ + ^16.5±11^	+ + ^3.7±3.0^	+ + + ^58.7±31^	+ + ^31.4±26^	*NP*	+ + ^17.5±6.5^	+ + + + ^44.1±20^
**3,7-Dimethyloct-2-ene** Isomer 2*^,S^		+ + + ^35.9±28^	+ + ^24.3±1.3^	+ + + + ^9.6±5.5^	+ + + + ^58.3±35^	+ + ^26.8±17^	*NP*	+ + + + ^21.4±20^	+ + + + ^45.4±14^
3,7-Dimethyloctan-1-ol		+ + ^3.9±3.7^	*NP*	+ + ^7.0±12^	+ + + ^6.1±3.8^	+ + ^6.1±10^	*NP*	+ + + ^3.6±5.0^	+ + ^1.9±2.5^
Limonene^S,L^		+ ^2.6±2.4^	*NP*	– ^0.2±0.3^	*NP*	+ ^3.2±4.9^	*NP*	+ + + + ^2.1±1.3^	+ + ^2.9±4.7^
(S)-Linalool^S,L^		+ + ^3.3±5.5^	*NP*	*NP*	*NP*	+ + + ^3.7±7.9^	*NP*	+ + + ^0.5±0.5^	+ + + + ^6.0±10^
*β*-Myrcene^S^		+ + ^3.5±1.9^	*NP*	+ + ^2.4±2.5^	*NP*	+ + + ^7.6±13^	*NP*	+ ^3.1±1.4^	*NP*
Nerol^S^		– ^3.1±5.4^	*NP*	*NP*	*NP*	+ + ^3.8±5.1^	*NP*	*NP*	*NP*
(*E*)-*β*-Ocimene^S^		*NP*	*NP*	+ + ^1.1±1.8^	*NP*	*NP*	*NP*	*NP*	*NP*
(*Z*)-*β*-Ocimene^S^		*NP*	*NP*	– ^2.2±3.8^	^++ 0.5±0.8^	*NP*	*NP*	*NP*	+ + ^2.0±3.3^
*Neoallo*-Ocimene^S^		*NP*	*NP*	– ^0.1±0.1^	*NP*	+ + + ^0.04±0.1^	*NP*	*NP*	*NP*
*α*-Pinene^L^		+ + ^4.4±10^	*NP*	– ^0.1±0.1^	*NP*	–^0.7±0.8^	*NP*	*NP*	*NP*
Sabinene^S^		*NP*	*NP*	*NP*	*NP*	*NP*	*NP*	+ ^1.2±1.0^	*NP*
*α*-Terpinene^S^		*NP*	*NP*	– ^1.1±0.9^	*NP*	+ + + ^0.1±0.3^	*NP*	*NP*	*NP*
*α*-Terpinolene^S^		+ ^2.1±2.8^	+ ^1.7±0.1^	*NP*	– ^1.4±0.9^	+ + ^0.9±0.8^	*NP*	+ + ^1.7±1.3^	*NP*
4-Terpinenol^S^		+ ^0.4±0.8^	*NP*	*NP*	*NP*	+ + ^0.5±1.0^	*NP*	*NP*	*NP*
*γ*-Terpinene^S^		– ^0.9±1.0^	*NP*	*NP*	*NP*	*NP*	*NP*	– ^0.5±0.6^	+ + + + ^0.3±0.5^
*Nitrogen-bearing compounds*									
Indole*^,S^		+ + ^220±117^	+ + ^273±203^	+ + + ^2.4±4.0^	+ + ^208±174^	+ + ^136±72^	+ + + ^79.2^	+ ^150±84^	+ + + + ^100±76^
*β*-Lutidine		+ + ^4.0±4.6^	*NP*	*NP*	*NP*	+ + + ^1.7±2.4^	+ + + ^2.1^	+ + ^2.1±2.5^	*NP*
**Skatole**^S^		+ + ^6.0±5.1^	+ + ^7.1±4.3^	– ^5.2 ± 5.2^	*NP*	+ ^2.8±2.2^	*NP*	– ^2.4±3.4^	*NP*
*Sesquiterpenoids*									
Bicyclogermacrene		+ + ^53.4±67^	*NP*	+ + + + ^20.3±13^	+ + + + ^91.5±117^	+ + + ^30.7±34^	+ + + ^25.9^	+ + + ^26.9±25^	+ + ^10.4±10^
*α*-Cadinene		*NP*	*NP*	– ^9.7±4.9^	*NP*	+ + + ^1.2±1.6^	*NP*	*NP*	*NP*
***δ*-Cadinene**		+ + ^35.0±27^	*NP*	+ + ^5.46±6.2^	*NP*	+ + + ^30.9±37^	+ + + ^2.6^	+ + + ^20.6±6.5^	*NP*
(*E*)-*β*-Caryophyllene*^,S^		+ + + ^46.4±32^	+ + + ^48.5±1.6^	+ + ^1.71±1.4^	+ + + + ^64.8±54^	+ + + ^77.4±68^	+ + + ^1.6^	+ + ^28.1±12^	+ + ^68.4±71^
*α*-Copaene^S^		+ + ^15.6±12^	+ + ^16.2±7.5^	– ^32.5±21^	+ + + ^19.8±21^	+ ^20.3±16^	+ + + ^0.9^	+ + ^8.60±3.2^	+ + ^18.1±17^
Germacrene D*^,S^		+ + + + ^48.2±50^	*NP*	*NP*	*NP*	+ + ^7.2±8.1^	*NP*	*NP*	*NP*
*α*-Humulene*^,S^		+ ^64.8±50^	+ ^70.5±4.2^	+ + + ^27.7±13^	+ + ^91.7±85^	– ^88.6±75^	*NP*	– ^41.0±17^	– ^75.7±78^
Isocaryophyllene		+ + ^20.5±15^	+ + ^22.4±5.4^	+ + ^0.5±0.8^	+ + ^26.9±26^	+ ^37.1±40^	*NP*	– ^13.3±5.6^	*NP*
*Unknowns*									
UNK 807	*m/z:* 70,98,42,71	+ ^3.5±1.5^	*NP*	*NP*	+ + ^4.01±1.0^	*NP*	*NP*	*NP*	*NP*
UNK 829	*m/z:* 84,67,110,41	+ ^3.3±3.0^	*NP*	+ + + ^43.3±28^	+ + + + ^3.7±0.5^	– ^5.1±8.2^	*NP*	*NP*	+ + ^1.4±2.3^
UNK 883	*m/z:* 79,77,108,91	+ ^0.3±0.6^	+ + + + ^0.5±0.8^	– ^0.5±0.4^	*NP*	*NP*	*NP*	*NP*	*NP*
UNK 962	*m/z:* 68,67,69,41	+ ^0.8±0.5^	*NP*	*NP*	*NP*	+++ ^0.9±1.2^	*NP*	*NP*	*NP*
UNK 1030	*m/z:* 95,67,138,82	+ + + ^4.9±5.8^	– ^1.6±1.3^	+ ^0.4±0.6^	+ + ^8.3±9.5^	+ + + ^2.2±2.0^	*NP*	+ + + + ^1.2±0.9^	+ + ^2.3±2.6^
**UNK 1135**	*m/z:* 82,67,59,121	*NP*	*NP*	*NP*	+ + + + ^0.9±0.5^	+ + + ^0.6±0.4^	*NP*	*NP*	+ + ^0.5±0.5^
UNK 1279	*m/z:* 43,68,81,54	*NP*	*NP*	+ + + + ^0.1±0.1^	*NP*	+ + + ^1.0±1.3^	*NP*	*NP*	*NP*
UNK 1349	*m/z:* 69,70,55,57	*NP*	*NP*	+ + + + ^1.9±1.4^	*NP*	+ + + ^0.03±0.0^	*NP*	*NP*	*NP*
UNK 1367_1	*m/z:* 81,95,55,93	+ + + ^5.1±3.8^	*NP*	+ + ^8.5±3.7^	*NP*	+ + + ^6.3±4.7^	*NP*	+ + + + ^5.2±3.7^	*NP*
**UNK 1378/1381**	*m/z:* 95,147,96,79/*m/z:* 55,69,95,81	+ + ^3.9±3.8^	+ ^3.4±2.3^	+ + ^0.5±0.5^	– ^8.0±4.0^	+ ^2.1±2.5^	+ + + ^0.5^	*NP*	*NP*
UNK 1394	*m/z:* 69,55,41,82	+ + ^13.1±15^	*NP*	*NP*	+ + + + ^23.2±23^	– ^11.1±21^	*NP*	*NP*	+ + ^22.1±24^
UNK 1409	*m/z:* 69,55,41,82	+ + ^0.8±1.4^	*NP*	+ + ^0.7±0.8^	*NP*	+ + + ^4.8±6.3^	*NP*	*NP*	*NP*
**UNK 1415**	*m/z:* 69, 81,41,95	+ + + ^119±80^	+ + + ^128±81^	+ + + ^0.6±0.7^	+ + ^209±51^	+ + + ^105±57^	+ + + ^6.2^	+ + + + ^102±63^	+ + + + ^118±63^
UNK 1466_1	*m/z:*161,107,122,105	+ + ^2.7±2.0^	*NP*	+ + ^0.9±0.9^	*NP*	+ + ^6.2±6.8^	*NP*	*NP*	*NP*
UNK 1492	*m/z:* 105,161,91,41	*NP*	*NP*	+ + ^22.9±15^	*NP*	– ^41.6±52^	*NP*	*NP*	*NP*
UNK 1492^‡‡^ and UNK 1503^‡‡^		+ + + ^14.1±17^	+ + + ^56.7±20^	*NP*	+ + + + ^17.1±22^	+ + ^56.7±20^	*NP*	+ + + ^32.9±6.9^	+ + + + ^33.0±57^
UNK 1503^‡^ and Germacrene D^S,‡^	*m/z*: 81,107,163	*NP*	*NP*	+ + + ^78.4±77^	*NP*	+ ^48.3±29^	*NP*	*NP*	*NP*
UNK 1518	*m/z:* 105,93,107,79	*NP*	*NP*	– ^0.4±0.5^	*NP*	+ + + ^2.6±4.8^	*NP*	*NP*	*NP*
UNK 1521_2	*m/z:*191,81,163,109	+ + ^8.5±9.9^	*NP*	*NP*	*NP*	*NP*	*NP*	+ + ^3.6±3.8^	*NP*
UNK 1541/ 1507_1	*m/z:*161,105,119,163	*NP*	+ ^19.1±2.2^	– ^18.5±32^	*NP*	+ + + ^6.1±11^	*NP*	+ + + ^10.4±4.2^	*NP*
UNK 1584	*m/z:* 85,57,41,53	+ + ^4.8±9.7^	*NP*	*NP*	*NP*	*NP*	*NP*	+ + + ^1.9±1.9^	*NP*
UNK 1607	*m/z:* 43,119,159,91	*NP*	*NP*	*NP*	*NP*	+ + ^0.5±1.0^	*NP*	*NP*	*NP*
UNK 1658	*m/z*:109,161,81,204	+ + + + ^5.8±3.8^	*NP*	*NP*	+ + + + ^6.0±1.6^	+ + + ^7.8±5.0^	*NP*	*NP*	*NP*
UNK 1699	*m/z:* 81,163,191,95	+ + ^112±90^	+ ^107±109^	*NP*	*NP*	+ + ^78.3±90^	*NP*	*NP*	*NP*

Levels of EAD-activity: +  +  +  + (EAD-active in > 80% of runs with samples that contained this compound), +  +  + (80–50%), +  + (< 50–25%), + (< 25%),—(VOC did not elicit a signal), *NP* (VOC not present in scent samples tested). * The physiological activity was confirmed by a synthetic standard (see Supplementary information Table S1); ^S^identity was confirmed by synthetic standard; ^L^in leaf blanks see^5^; m/z = mass-to-charge ratio in decreasing order of abundance; ^‡^coeluted on non-chiral ZB-5 column; ^‡‡^coeluted on chiral columns.

Electrophysiological measurements were performed with a gas chromatograph (Agilent 7890A, Santa Clara, California, USA) equipped with a flame ionization detector (FID) and an electroantennographic detection system (GC–EAD)^44,49^. One microliter of a solvent scent sample was injected in splitless mode (250 °C), with hydrogen as the carrier gas (column flow: 3 mL min^−1^). During the period of testing (2017–2020), the GC was equipped with three different columns. In 2017, it was a ZB-5 fused silica column (5% phenyl polysiloxane; 30 × 0.32 mm, 0.25 µm film thickness; Phenomenex, Torrance, CA, USA), which was replaced in 2018 by a chiral fused silica capillary (30 m × 0.23 mm I.D.), coated with a 0.23 μm film of 0.4% heptakis (2,3-di-O-methyl-6-O-tert-butyldimethylsilyl)-*β*-cyclodextrin (DIME-*β*-CD) (30%) in SE-52 (70%), the same as described in^52^ and^53^. From mid-2019 onwards, the GC was equipped with another DIME-*β*-CD chiral column (MEGA-DEX DMT Beta SE, 30 m × 0.25 mm ID, 0.23 µm film thickness, MEGA S.r.l., Legnano, Italy). The end of each column was split into two capillaries by a μFlow splitter (Gerstel, Mühlheim, Germany), with nitrogen (N_2_) as make-up gas (flow rate 25 mL min^−1^). One of the capillaries (2 m × 0.15 µm inner diameter) led to the FID and the other (1 m × 0.2 µm inner diameter) to the EAD setup. The EAD was set up by a transfer line, heated at 220 °C, and a 2-channel USB acquisition controller (Syntech, Kirchzarten, Germany). The outlet of the EAD was placed in a cleaned, humidified airflow, directed onto the mounted antenna. Prior to measurements, each fly was anaesthetised with CO_2_, and the head and last antennomere (apical-tip) were removed. Subsequently, the head and one randomly selected antenna were each connected to a glass micropipette electrode, filled with 95% insect Ringer’s solution (8.0 g L^−1^ NaCl, 0.4 g L^−1^ KCl, 4.0 g L^−1^ CaCl_2_) and 5% Tween 20 (Sigma Aldrich, Vienna, Austria), and connected to silver wires. The recording electrode was attached to the tip of the antenna, while the reference electrode was connected to the caudal side of the head^44,54^.

Solvent scent samples of *A. maculatum* were tested on the antennae of five Diptera species: the Sphaeroceridae *Coproica ferruginata* stenh. (one female) and four Psychodidae species*, i.e., Psychoda phalaenoides* (12 females and eight males), *P. zetterstedti* (two females and two males), *P. trinodulosa* (one male), and *P. cinerea* banks (two females) (Table 1, Supplementary information Table S1). All these fly species are visitors of *A. maculatum*^24,25^^;^^27^, except for *P. cinerea,* which is a pollinator of *A. hygrophilum* and *A. italicum*^29^, with the latter sharing several floral compounds with *A. maculatum*^21,32^. Two additional female *Psychoda* individuals (collected directly from *A. maculatum*) could not be determined to species level, as their abdomens were too damaged, and might belong to the four above mentioned species or (an)other species.

For identification of EAD-active compounds, scent samples were run on a gas chromatograph/mass spectrometer (GC/MS, model QP2010 Ultra EI, Shimadzu, Tokyo, Japan), equipped with either a non-chiral ZB-5 column (in 2017; see above) or a chiral column (2018–2020; MEGA-DEX DMT Beta SE, see above). Helium was used as carrier gas (flow: 3 mL min^−1^) and samples (injection volume: 1 µL) were run with a split ratio of 1:1^44,49^. Obtained data were handled using *GCMSolution* v.4.41 (Shimadzu Corporation, Kyoto, Japan). We tentatively identified components by comparison of Kováts’ retention indices^55^ (KRIs; based on commercially available *n*-alkanes C_7_–C_20_) and mass spectra available in the libraries of Adams^56^, FFNSC 2, Wiley9, NIST11, and ESSENTIAL OILS (available in *MassFinder 3,* Hochmuth Scientific Consulting, Hamburg, Germany). The identity of some of the components was verified by authentic reference standards, available in the collection of the Plant Ecology Lab of Salzburg University (see Table S1). Compounds were classified as inflorescence-specific or as vegetative compounds, according to Gfrerer et al.^5^. Absolute amounts of compounds tested in the GC/EAD measurements (Table 1) were quantified by injecting known amounts of various aliphatics and terpenoids and the resulting mean peak areas were used for quantification^5^.

## Results

**Figure 2:**
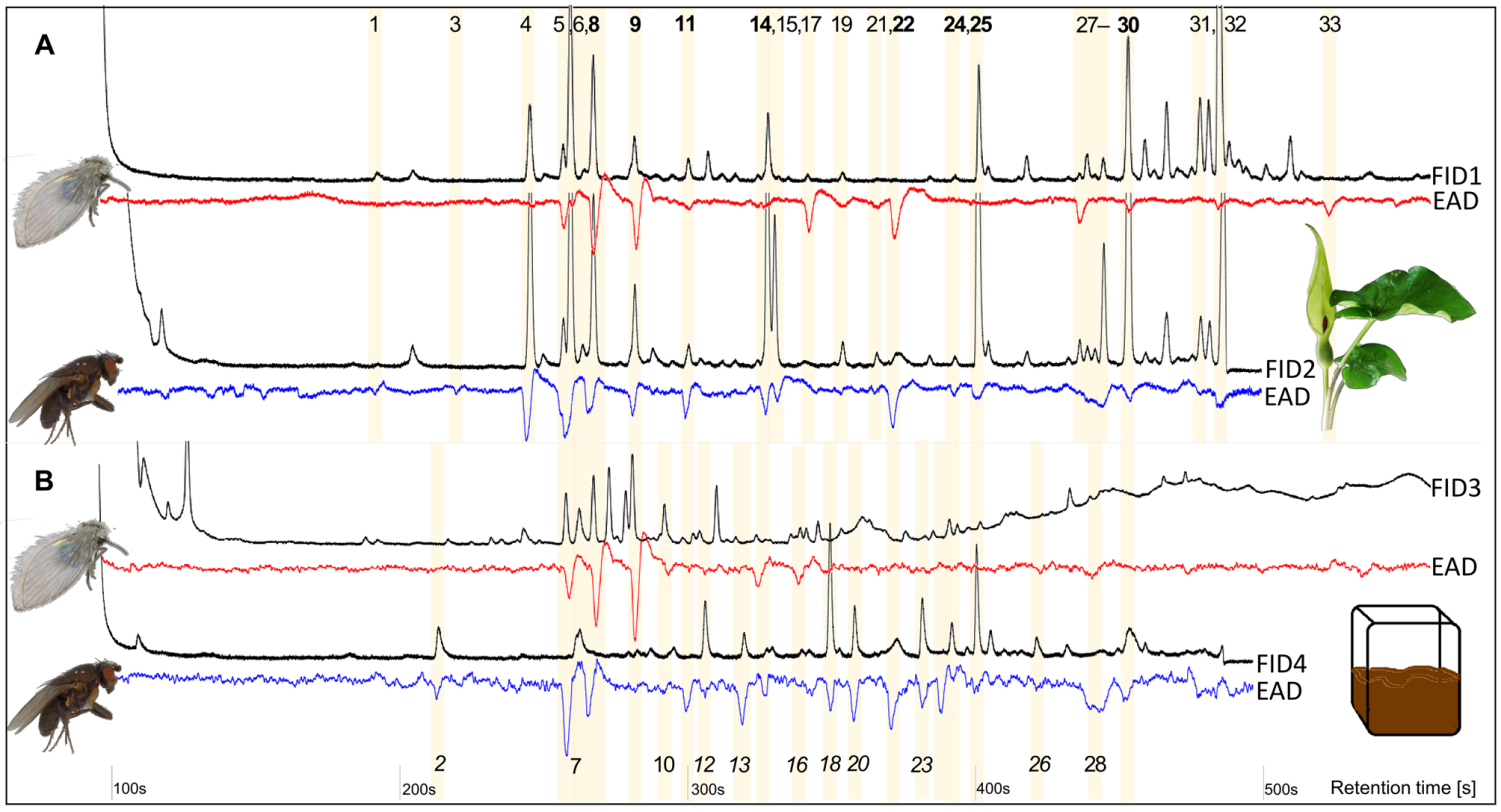
Representative physiological responses (gas chromatography coupled to electroantennographic detection) of a female *Psychoda* sp*.* (Psychodidae; red) and a female *Coproica ferruginata* (Sphaeroceridae; violet) to scent samples of (**A**) inflorescences of *Arum maculatum* and (**B**) cow dung (fresh and 1-day old). EAD-active compounds (see also Table 1): (1) UNK883; (2) hexanal; (3) 1-hexanol; (4) 3,7-dimethyloct-1-ene; (5) ( +)-*α*-citronellene; (6) ( +)-*β*-citronellene; (7) 3,7-dimethyl-2-octene isomer 1; (8) 3,7-dimethyl-2-octene isomer 2; (9) 2,6-dimethyl-2,6-octadiene isomer 2; (10) 6-methyl-5-hepten-2-one; (11) UNK1030; (12) octanal; (13) *p*-cymene; (14) 6-methyl-5-heptene-2-ol; (15) 1-octen-3-ol; (16) 2-methylbutanoic acid; (17) 2-nonanone; (18) nonanal; (19) 2,6-dimethyl-7-octen-2-ol; (20) (*E*)-2-octen-1-ol; (21) (*S*)-linalool; (22) 2-decanone; (23) UNK1135; (24) decanal; (25) *p*-cresol; (26) 3,7-dimethyl-1-octanol; (27) *α*-copaene; (28) UNK1378/81; (29) UNK1394; (30) UNK1415; (31) indole; (32) UNK1503/UNK1492; (33) UNK 1658. Numbers in bold (top line) are VOCs eliciting signals in (**A**) and (**B**); numbers in italics (bottom line) are VOCs that do not occur in *A. maculatum*. FID1 *A. maculatum,* population Josefiau; FID2 *A. maculatum,* population Murnau; FID3 cow dung fresh; FID4 cow dung 1-day-old. See Supplementary Table S1 for detailed population information. All samples shown were run on a chiral fused silica capillary column (30% DIME-*β*-CD in 70% SE-52, see methods) and measurements lasted either 8 (*Coproica ferruginata*) or 10 min (*Psychoda* sp.).

Across all tested dipterans, we found a total of 78 volatile organic compounds (VOCs) from *A. maculatum* (together c. 88% of the relative inflorescence scent emission of this species^5^) that were electroantennographically active. The majority of these compounds were inflorescence-specific, but five of them were vegetative compounds [*i.e.,* (*Z*)-3-hexenyl acetate, nonanal, limonene, linalool, *α*-pinene; Table 1]. Overall, 55 of the EAD-active VOCs could be (tentatively) identified (Table 1). They represented several chemical classes, including monoterpenoids (*n* = 25 VOCs), aliphatic compounds (15), sesquiterpenoids (eight), irregular terpenoids (two), nitrogen-bearing compounds (three), and aromatic compounds (two). Seven of these VOCs elicited antennal responses in all tested insect species: the monoterpenoid 2,6-dimethylocta-2,6-diene (isomer 2), the aliphatic compound 1-octen-3-ol, the sesquiterpene (*E*)-*β*-caryophyllene, the nitrogen-containing component indole, the aromatic components methyl salicylate and *p*-cresol, and the unknown UNK1415 (Table 1, Fig. 2). All other VOCs elicited responses only in a subset of insect species.

On average, 38 scent compounds yielded a response per species (and sex), with a minimum of 13 volatiles in male *P. trinodulosa* (Psychodidae; one individual tested on one scent sample), and a maximum of 60 VOCs in female *P. phalaenoides* (12 individuals tested on nine scent samples). When considering both sexes of *P. phalaenoides* (total of 20 individuals tested on 16 scent samples), 61 VOCs were EAD-active, and in both sexes of *P. zetterstedti* 49 VOCs (four individuals on eight scent samples; Table 1, Supplementary information Table S1). For the female *C. ferruginata* (Sphaeroceridae; one individual tested on three samples), 36 VOCs resulted in an antennal response. Notably, the aliphatic compound 2-nonanone was perceived by all psychodid species, but did not elicit signals in *C. ferruginata*, even though this compound was present in all three scent samples tested on the latter species. In contrast, the monoterpene *γ*-terpinene was EAD-active only in *C. ferruginata*, but not in the two psychodid species tested on this compound, *i.e*., *P. phalaenoides* and *P. sp.*

Some compounds elicited specific responses in certain *Psychoda* taxa. For instance, the aliphatic compound nonanal induced responses in *P. phalaenoides* and *P. zetterstedti*, but not in *P. cinerea* and *P. trinodulosa*. The nitrogen-bearing compound skatole was EAD-active in *P. phalaenoides* and *P. cinerea,* but not in *P. zetterstedti* and *P. sp.* (Table 1). The sesquiterpene *α*-humulene resulted in responses in most individuals of *P. zetterstedti* and in a few individuals of *P. phalaenoides*, but not in *P. cinerea* and *P. sp*. A number of the unknown volatiles (*e.g.*, UNK883, UNK1394, UNK1492) elicited responses in *P. phalaenoides* and *P. zetterstedti*, but not in *P. cinerea*.

For *P. phalaenoides* and *P. zetterstedti*, the two species for which we tested male and female individuals, the analyses revealed some sex-specific responses. In *P. phalaenoides,* 24 compounds were EAD-active in both sexes, but one compound elicited responses only in females (unknown UNK1030; *n* = 12 individuals). In *P. zetterstedti*, 21 compounds were EAD-active in both sexes, but four compounds [2,6-dimethylocta-2,6-diene isomer 1, (*Z*)-*β*-ocimene, *α*-copaene, 6-methyl-5-hepten-2-one] and two [**( +)-***α*-citronellene, unknown UNK1378/81] elicited responses only in females (*n* = 2) and males (*n* = 2), respectively.

Of the 78 EAD-active floral compounds, 18 were also physiologically active in cow dung samples (Fig. 2, Table 1), representing all different chemical classes recorded in *A. maculatum*. More specifically, six of the seven VOCs that elicited antennal responses in all tested insect species were among those 18 compounds [*i.e.,* 2,6-dimethylocta-2,6-diene isomer 1, 1-octen-3-ol, (*E*)-*β*-caryophyllene, indole, *p*-cresol, and UNK1415; Fig. 2]. Among those 18 were also VOCs that elicited different responses among fly families (2-nonanone), fly species (nonanal, skatole), and between sexes within species [*i.e.,* 2,6-dimethylocta-2,6-diene isomer 1, 6-methyl-5-hepten-2-one, (*E*)-*β*-caryophyllene].

## Discussion

Our study is the first to identify electroantennographically active compounds in *Psychoda* spp. (Psychodidae) and a Sphaeroceridae (*Coproica ferruginata*). It shows that these insect visitors of deceptive *Arum maculatum* are sensitive to a high number of the plants’ inflorescence scent compounds. The EAD-active compounds identified represent various chemical classes, including mono- and sesquiterpenoids, aliphatic, aromatic, nitrogen-bearing, and unknown compounds. Antennal responses differed among insect species and between sexes within species. More than a fifth of the physiologically active scent compounds were also released by cow dung, linking insect breeding/mating sites, floral VOCs of *A. maculatum*, and its floral visitors.

A few of the compounds recorded as physiologically active in the antennae of *Psychoda* spp. (1-octen-3-ol, butanoic acid, *α*-pinene, and *α*-terpinene) were also found to elicit electrophysiological responses in females of the phlebotomine sandfly *Lutzomyia longipalpis* (Psychodidae, Diptera)^56,57^, the only other psychodid used so far for physiological measurements in the olfactory circuitry. This sandfly, which was tested on faeces from vertebrates and canid host odours, additionally responded to several other volatiles that do not occur in *A. maculatum* (including different isomers of monoterpenoids), but we provide the first evidence that psychodids are able to perceive sesquiterpenoids. Physiological measurements on antennae of Sphaeroceridae were not available before our measurements, and thus overall, our study increases the knowledge about the peripheral olfactory circuitry of psychodids and Sphaeroceridae.

Among the EAD-active volatiles recorded in the present study are the most abundant inflorescence scents of *A. maculatum* included (*i.e.,* indole, *p*-cresol, **( +)-***β*-citronellene, 2-heptanone, 3,7-dimethyloct-2-ene, UNK1415^5,19,21–23,32^), but also numerous compounds emitted only in small relative amounts by the plant (*e.g.,* 3-octanone, 2,6-dimethyl-7-octen-2-ol; Fig. 2). Several of these abundant and less abundant compounds are also released from cow dung (Table 1, Fig. 2) or other breeding/mating sites of the tested insect species. These are, for instance, *p*-cresol, 2-heptanone, terpinen-4-ol, *α*-citronellene, and 2,6-dimethylocta-2,6-diene (cow dung; Table 1^40^), 2-octanone (horse dung^39^), *β*-citronellene and *α***-**humulene (both cow and horse dung^39,40,59^), or 3-octanone and (*E*)-2-octen-1-ol (fungi^60^). Some of the EAD-active compounds have not been detected in ours or others’ scent samples from (cow) dung and have not been described elsewhere. We speculate that those volatiles are released from various other, likely differently scented breeding and/or mating substrates of *A. maculatum* pollinators, such as mud-flats or leaf litter. It has been suggested that the hyperdiverse floral scent of *A. maculatum* might result from the imitation of various differently scented breeding substrates^5^. The sensitivity of the tested flies to a variety of different compounds would support this idea, although other possible roles for those volatiles cannot be excluded (*e.g*., repellence of florivores^61^, defence against pathogens^62^).

Altogether five inflorescence scent compounds of *A. maculatum* have previously been reported as attractive to psychodids. In detail, indole and *p*-cresol, together with *α*-humulene or 2-heptanone, were found to attract female *P. phalaenoides*^41^ and *Psychoda* spp.^34^. In Kite *et al*.^34^, the attracted psychodids have not been identified to the species level; hence, it is unknown whether they are flower visitors of *A. maculatum* or not. A synthetic mixture of skatole, indole, and *p*-cresol, together with VOCs not occurring in *A. maculatum* (geranyl acetone, dihydro-, and *β*-ionone), was shown to attract psychodid and sphaerocerid pollinators of *Typhonium eliosurum*, a dung-mimicking aroid endemic to Australia^28^. In preliminary bioassays in the field, we tested the above five compounds, using the same composition and concentration as released by the inflorescences of *A. maculatum*. Yet those volatiles did not attract psychodid or sphaerocerid flies. This suggests that other, not yet tested scent compounds (additionally) contribute to pollinator attraction in *A. maculatum*. Potential candidates are other odours known also from cow or horse dung (*e.g.,* 2,6-dimethylocta-2,6-diene, unknown UNK1415; Table 1) or compounds known from other breeding substrates (*e.g*., fungi: 3-octanone^60^). The unknown UNK1415, one of the main scent compounds of *A. maculatum*^5^, yielded antennal responses in all insect taxa and in nearly all individuals tested in the present study. Interestingly, this unknown volatile is possibly identical to unknown “RI 1531” found in *T. eliosurum*^28^, as both volatiles have the same mass spectra (Supplementary information Fig. S1).

Our study shows that some of the antennal responses to scent differ among insect species, and some also between males and females within species. This finding is in agreement with results obtained by physiological measurements in other insects^43,63^. Some of the species-level effects described in the present study might have been influenced by sex-specific effects, because for some species (*P. cinerea* and *P. trinodulosa*) we only tested males or females. Hence, differences in antennal responses among these species need to be interpreted with caution. Nonetheless, species- and sex-specific differences in the peripheral olfactory circuitry of insects can result in different behaviours^64,65^. Interestingly, antennae of *P. cinerea*, the only non-pollinating species of *A. maculatum* (but of other *Arum* spp.) we tested in this study, did not respond to some abundant compounds emitted by *A. maculatum* (*e.g.,* UNK1394, UNK1492), which otherwise elicited responses in the other pollinating species tested (*e.g*., *P. phalaenoides* and *P. zetterstedti*). The lack of antennal sensitivity to (some of) those compounds might explain why *P. cinerea* does not visit *A. maculatum*, while close relatives including *P. phalaenoides* and *P. zetterstedti* are (important) pollinators^24,25^.

## Conclusions

Until now, it was not known which (and how many) volatile compounds of the complex floral scent of *Arum maculatum* can be perceived by its floral visitors. Our study identified 78 physiologically active compounds from hundreds of potentially behaviourally active VOCs, which is still a rather high number the psychodid and sphaerocerid flies are sensitive to. Our results thus provide a basis for future studies that aim to understand the floral volatiles of *A. maculatum* involved in the chemical attraction and deception of its pollinators, and which VOCs guide the flies to their breeding/mating substrates. Some of the EAD-active VOCs (4-terpinenol, *α*-terpinene, 2-heptanol, 2-nonanol, UNK1503) have recently been shown to be under phenotypic selection in *A. maculatum*^5^. These compounds and those EAD-active ones shared with the pollinators’ breeding substrates (*e.g.,* UNK1415, 3-octanone) are the most promising candidates for future behavioural assays. As the tested Diptera species (Psychodidae, Sphaeroceridae) are also known pollinators of other (similarly-scented) species of *Arum* (*e.g., A. italicum, A. concinnatum*^31, 66^) as well as other species/genera of Araceae (*e.g*., *Typhonium eliosurum*^28^), our study should also help to elucidate the chemical interactions between these plants and their fly pollinators. Future research is now needed to test the behavioural function of physiologically active floral volatiles, which is crucial for a better understanding of olfactory cues mediating plant–animal interactions in general, and in sapromyiophilous species, in particular.

## Experimental research and field studies on plants

All samplings were carried out in compliance with the current laws of the respective countries.

## Supplementary Information

Supplementary Information.

## Data Availability

All data that support the findings of this study are included in this published article (and its supplementary information files).

## References

[1] Raguso RA (2008). Wake up and smell the roses: the ecology and evolution of floral scent. Annu. Rev. Ecol. Evol. Syst..

[2] Knudsen JT, Eriksson R, Gershenzon J, Ståhl B (2006). Diversity and distribution of floral scent. Bot. Rev..

[3] Hadacek F, Weber M (2002). Club-shaped organs as additional osmophores within the *Sauromatum* inflorescence: odour analysis, ultrastructural changes and pollination aspects. Plant Biol..

[4] Schlumpberger BO, Raguso RA (2008). Geographic variation in floral scent of *Echinopsis ancistrophora* (Cactaceae); evidence for constraints on hawkmoth attraction. Oikos.

[5] Gfrerer, E. *et al.* Floral scents of a deceptive plant are hyperdiverse and under population-specific phenotypic selection. *Front. Plant Sci.* **12**, https://doi.org/10.3389/fpls.2021.719092 (2021). 10.3389/fpls.2021.719092 PMC8500232 34630465

[6] Primante C, Dötterl S (2010). A syrphid fly uses olfactory cues to find a non-yellow flower. J. Chem. Ecol..

[7] Knauer AC, Schiestl FP (2015). Bees use honest floral signals as indicators of reward when visiting flowers. Ecol. Lett..

[8] Theis N (2006). Fragrance of Canada thistle (*Cirsium arvense*) attracts both floral herbivores and pollinators. J. Chem. Ecol..

[9] Bouwmeester H, Schuurink RC, Bleeker PM, Schiestl F (2019). The role of volatiles in plant communication. Plant J..

[10] Schiestl FP (1999). Orchid pollination by sexual swindle. Nature.

[11] Schäffler I (2015). Diacetin, a reliable cue and private communication channel in a specialized pollination system. Sci. Rep..

[12] Castañeda-Zárate M, Johnson SD, van der Niet T (2021). Food reward chemistry explains a novel pollinator shift and vestigialization of long floral spurs in an orchid. Curr. Biol..

[13] Dötterl S, David A, Boland W, Silberbauer-Gottsberger I, Gottsberger G (2012). Evidence for behavioral attractiveness of methoxylated aromatics in a dynastid scarab beetle-pollinated Araceae. J. Chem. Ecol..

[14] Maia ACD (2012). The key role of 4-methyl-5-vinylthiazole in the attraction of scarab beetle pollinators: a unique olfactory floral signal shared by Annonaceae and Araceae. J. Chem. Ecol..

[15] Stamm P, Etl F, Maia ACD, Dötterl S, Schulz S (2021). Synthesis, absolute configurations, and biological activities of floral scent compounds from night-blooming Araceae. J. Org. Chem..

[16] Jürgens A, Wee SL, Shuttleworth A, Johnson SD (2013). Chemical mimicry of insect oviposition sites: a global analysis of convergence in angiosperms. Ecol. Lett..

[17] Zito P, Sajeva M, Raspi A, Dötterl S (2014). Dimethyl disulfide and dimethyl trisulfide: so similar yet so different in evoking biological responses in saprophilous flies. Chemoecology.

[18] El-Sayed, A. M. The Pherobase: database of pheromones and semiochemicals. https://www.pherobase.com (2021).

[19] Kite GC (1995). The floral odour of *Arum maculatum*. Biochem. Syst. Ecol..

[20] Chartier M, Pélozuelo L, Gibernau M (2011). Do floral odor profiles geographically vary with the degree of specificity for pollinators? Investigation in two sapromyophilous *Arum* species (Araceae). Ann. Soc. Entomol. Fr..

[21] Chartier M, Pélozuelo L, Buatois B, Bessière JM, Gibernau M (2013). Geographical variations of odour and pollinators, and test for local adaptation by reciprocal transplant of two European *Arum* species. Funct. Ecol..

[22] Marotz-Clausen, G. *et al.* Incomplete synchrony of inflorescence scent and temperature patterns in *Arum maculatum* L. (Araceae). *Phytochemistry* **154**, 77–84 (2018). 10.1016/j.phytochem.2018.07.001 30006091

[23] Szenteczki MA (2021). Spatial and temporal heterogeneity in pollinator communities maintains within-species floral odour variation. Oikos.

[24] Espíndola A, Pellissier L, Alvarez N (2011). Variation in the proportion of flower visitors of *Arum maculatum* along its distributional range in relation with community-based climatic niche analyses. Oikos.

[25] Laina, D. *et al.* Local insect availability partly explains geographical differences in floral visitor assemblages of *Arum maculatum* L. (Araceae). *Front. Plant Sci.* **13**, https://doi.org/10.3389/fpls.2022.838391 (2022). 10.3389/fpls.2022.838391 PMC8957888 35350299

[26] Tonnoir, A. L. A synopsis of the British Psychodidae (Dipt.) with descriptions of new species. *Trans. Soc. Br. Entomol.* **7**, 21–64 (1940).

[27] Roháček J, Beck-Haug I, Dobat K (1990). Sphaeroceridae associated with flowering *Arum maculatum* (Araceae) in the vicinity of Tübingen, SW-Germany (Insecta: Diptera). Senckenb. Biol..

[28] Sayers TDJ, Steinbauer MJ, Farnier K, Miller RE (2020). Dung mimicry in *Typhonium* (Araceae): explaining floral trait and pollinator divergence in a widespread species complex and a rare sister species. Bot. J. Linn. Soc..

[29] Gibernau M, Macquart D, Przetak G (2004). Pollination in the genus *Arum*: a review. Aroideana.

[30] Kakishima, S. & Okuyama, Y. Pollinator assemblages of *Arisaema heterocephalum* subsp. *majus* (Araceae), a critically endangered species endemic to Tokunoshima Island, Central Ryukyus. *Bull. Natl. Mus. Nat. Sci., Ser. B* **44**, 173–179 (2018).

[31] Urru I (2010). Pollination strategies in Cretan *Arum* lilies. Biol. J. Linn. Soc..

[32] Diaz A, Kite GC (2002). A comparison of the pollination ecology of *Arum maculatum* and *Arum italicum* in England. Watsonia.

[33] Lack, A. J. & Diaz, A. The pollination of *Arum maculatum* L.: a historical review and new observations. *Watsonia* **18**, 333–342 (1991).

[34] Kite, G. C. *et al.* Inflorescence odours and pollinators of *Arum* and *Amorphophallus* (Araceae). in *Reproductive Biology* (eds. Owens, S. J. & Rudall, P. J.) 295–315 (Kew Royal Botanic Gardens, 1998).

[35] Laurence BR (1954). The larval inhabitants of cow pats. J. Anim. Ecol..

[36] Wagner R (1977). Zur Kenntnis der Psychodidenfauna des Allgäus. Nachrichtenblatt der Bayer. Entomol..

[37] Satchell GH (1947). The ecology of the British species of *Psychoda* (Diptera: Psychodidae). Ann. Appl. Biol..

[38] Withers P, O’Connor JP (1992). A preliminary account of the Irish species of moth fly (Diptera: Psychodidae). Proc. R. Ir. Acad. B..

[39] Dormont L, Jay-Robert P, Bessière JM, Rapior S, Lumaret JP (2010). Innate olfactory preferences in dung beetles. J. Exp. Biol..

[40] Sládeček FXJ, Dötterl S, Schäffler I, Segar ST, Konvicka M (2021). Succession of dung-inhabiting beetles and flies reflects the succession of dung-emitted volatile compounds. J. Chem. Ecol..

[41] Scheven, H. J. GC/MS Untersuchungen des Appendixduftes blühender Pflanzen von *Arum maculatum* L. und *Arum italicum* MILLER; Nachweis der attraktiven Wirkung der Duftbestandteile Indol, Humulen und p-Kresol auf *Psychoda phalaenoides* L. (Philipps-Universität Marburg, 1994).

[42] Schiestl, F. P. & Marion-Poll, F. Detection of physiologically active flower volatiles using gas chromatography coupled with electroantennography. in *Analysis of Taste and Aroma* (eds. Jackson, J. F. & Linskens, H. F.) 173–198 (Springer Berlin Heidelberg, 2002).

[43] Jhumur US, Dötterl S, Jürgens A (2007). Electrophysiological and behavioural responses of mosquitoes to volatiles of *Silene otites* (Caryophyllaceae). Arthropod. Plant. Interact..

[44] Heiduk A (2016). *Ceropegia sandersonii* mimics attacked honeybees to attract kleptoparasitic flies for pollination. Curr. Biol..

[45] Suinyuy, T. N., Donaldson, J. S. & Johnson, S. D. Geographical matching of volatile signals and pollinator olfactory responses in a cycad brood-site mutualism. *Proc. R. Soc. B Biol. Sci.* **282**, (2015). http://doi.org/10.1098/rspb.2015.2053 10.1098/rspb.2015.2053 PMC4614789 26446814

[46] Dötterl S (2005). Nursery pollination by a moth in *Silene latifolia*: The role of odours in eliciting antennal and behavioural responses. New Phytol..

[47] Schiestl FP (2003). The chemistry of sexual deception in an orchid-wasp pollination system. Science.

[48] Stensmyr MC (2002). Rotting smell of dead-horse arum florets. Nature.

[49] Lukas K, Harig T, Schulz S, Hadersdorfer J, Dötterl S (2019). Flowers of European pear release common and uncommon volatiles that can be detected by honey bee pollinators. Chemoecology.

[50] Bermadinger-Stabentheiner E, Stabentheiner A (1995). Dynamics of thermogenesis and structure of epidermal tissues in inflorescences of *Arum maculatum*. New Phytol..

[51] Dötterl S, Füssel U, Jürgens A, Aas G (2005). 1,4-Domethoxybenzene, a floral scent in willows that attracts an oligolectic bee. Journal of Chemical Ecology.

[52] Dötterl, S. *et al.* Linalool and lilac aldehyde/alcohol in flower scents. Electrophysiological detection of lilac aldehyde stereoisomers by a moth. *J. Chromatogr. A* **1113**, 231–238 (2006). 10.1016/j.chroma.2006.02.011 16542668

[53] Brandt K (2019). Subtle chemical variations with strong ecological significance: stereoselective responses of male orchid bees to stereoisomers of carvone epoxide. J. Chem. Ecol..

[54] Zito P, Dötterl S, Sajeva M (2015). Floral volatiles in a sapromyiophilous plant and their importance in attracting house fly pollinators. J. Chem. Ecol..

[55] Kováts E, Weisz P (1965). Über den Retentionsindex und seine Verwendung zur Aufstellung einer Polaritätsskala für Lösungsmittel. Berichte der Bunsengesellschaft für Phys. Chem..

[56] Dougherty MJ, Guerin PM, Ward RD, Hamilton JGC (1999). Behavioural and electrophysiological responses of the phlebotomine sandfly *Lutzomyia longipalpis* (Diptera: Psychodidae) when exposed to canid host odour kairomones. Physiol. Entomol..

[57] Sant’Ana, A. L., Eiras, A. E. & Cavalcante, R. R. Electroantennographic responses of the *Lutzomyia (Lutzomyia) longipalpis* (Lutz and Neiva) (Diptera: Psychodidae) to 1-octen-3-ol. *Neotrop. Entomol.* **31**, 13–17 (2002).

[58] Adams, R. P. *Identification of essential oil components by gas chromatography/mass spectrometry*. (Allured Publishing Corporation, 2007).

[59] Johnson SD, Jürgens A (2010). Convergent evolution of carrion and faecal scent mimicry in fly-pollinated angiosperm flowers and a stinkhorn fungus. S. Afr. J. Bot..

[60] Thakeow P, Angeli S, Weißbecker B, Schütz S (2008). Antennal and behavioral responses of *Cis boleti* to fungal odor of *Trametes gibbosa*. Chem. Senses.

[61] Junker RR, Blüthgen N (2010). Floral scents repel facultative flower visitors, but attract obligate ones. Ann. Bot..

[62] Junker RR, Tholl D (2013). Volatile organic compound mediated interactions at the plant-microbe interface. J. Chem. Ecol..

[63] Abraham J (2015). Behavioral and antennal responses of *Drosophila suzukii* (Diptera: Drosophilidae) to volatiles from fruit extracts. Environ. Entomol..

[64] Stökl J (2008). Scent variation and hybridization cause the displacement of a sexually deceptive orchid species. Am. J. Bot..

[65] Salamanca J, Souza B, Lundgren JG, Rodriguez-Saona C (2017). From laboratory to field: electro-antennographic and behavioral responsiveness of two insect predators to methyl salicylate. Chemoecology.

[66] Revel N, Alvarez N, Gibernau M, Espíndola A (2012). Investigating the relationship between pollination strategies and the size-advantage model in zoophilous plants using the reproductive biology of *Arum cylindraceum* and other European *Arum* species as case studies. Arthropod. Plant. Interact..

