# Supplementary material for: Antennae of psychodid and sphaerocerid flies respond to a high variety of floral scent compounds of deceptive Arum maculatum L
Source: Sci Rep. 2022 Mar 24;12:5086. doi: 10.1038/s41598-022-08196-y (PMC8948215; doi:10.1038/s41598-022-08196-y)
Supplement: Supplementary file 1 — Supplementary Information. [file 41598_2022_8196_MOESM1_ESM.pdf]

Supplementary Information

Title: Antennae of psychodid and sphaerocerid flies respond to a high variety of floral scent compounds of deceptive *Arum maculatum* L.

Authors: Gfrerer Eva; Laina Danae; Wagner Rüdiger; Gibernau Marc; Hörger Anja C.; Comes Hans Peter; Dötterl Stefan

Available Information:

Fig S1. Mass spectrum of unknown compound UNK1415.

Table S1. Number of EAD-runs per fly species and origin of tested scent samples.

Fig S1. Mass spectrometry from unknown compound UNK1415.

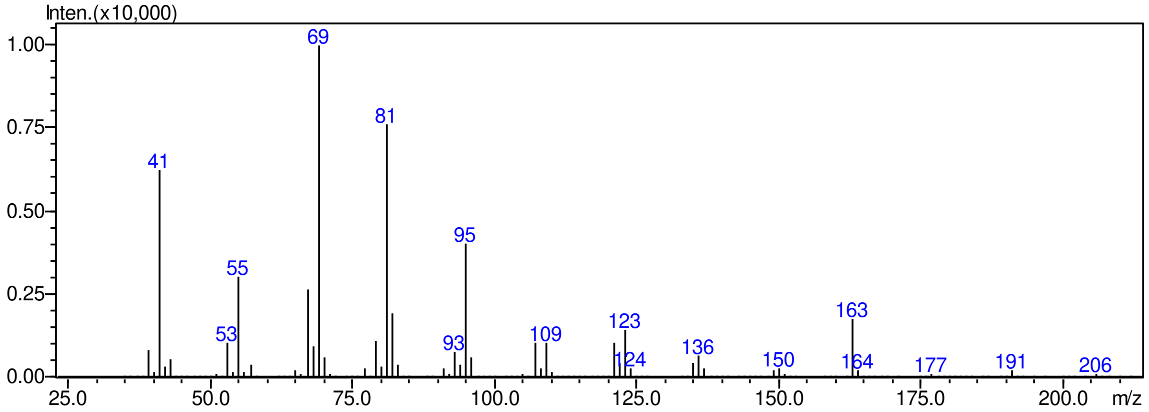

Table S1: Number of EAD-runs per fly species (Psychodidae and Sphaeroceridae, Diptera) and individual, geographic origin of tested scent samples, and volatile organic compounds (VOCs) used for synthethic mixtures.

‡ synthetic volatiles, in highest purity available (supplier: Sigma-Aldrich, Germany) or chemically synthesised (see Gfrerer *et al*., 2021, *Frontiers in Plant Science*).

§ not included in EAD Table, as they are not released by *Arum maculatum*

		Number of individuals/ runs			Origin of natural scent samples							
**Species**	**f/m**	**# individuals tested on natural/ synthetic samples**	**# runs performed with natural/ synthetic samples**	**# of inflorescence/ synthetic scent samples used**	**North of the Alps**				**South of the Alps**			
					**JOS**	**MUR**	**NEC**	**RÜM**	**DAO**	**LIM**	**MAH**	**MON**
Psychoda phalaenoides	f	12/9	33/19	9/6		x			x			
P. phalaenoides	m	8/2	14/3	3/2	x	x	x		x		x	
P. zetterstedti	f	2/2	6/5	4/1	x				x			x
P. zetterstedti	m	2/2	3/3	3/0	x	x			x			
P. cinerea	f	2/2	6/6	5/0	x	x			x	x		
P. sp.	f	2/2	8/5	4/3	x		x		x		x	
P. trinodulosa	m	1/1	1/1	1/0				x				
Coproica ferruginata	f	1/1	3/3	3/0	x				x			
Country					Austria	Germany	Germany	Switzerland	Italy	Italy	Italy	Italy
Location					Salzburg (Josefiau)	Murnau am Staffelsee	Horb am Neckar	Rümikon/ Aargau	Daone/ Trento	Limone Piemonte	S. Maria Hoè/ Lecco	Montese/ Modena
Latitude (N)					47°46.98’	47°40.20’	48°25.20’	47°31.00’	45°57.60’	44°12.60’	45°44.90’	44°16.29’
Longitude (E)					13°04.50’	11°10.80’	8°39.00’	8°21.00’	10°34.80’	7°34.20’	9°22.00’	10°56.42’

		Synthetic mixtures							VOCs‡
**Species**	**f/m**	**Mix_N1**	**Mix_N2**	**Mix_S1**	**Mix_S2**	**Mix_3**	**Mix_4**	**Mix_5**	
		10^{-4}	10^{-4}	10^{-4}	10^{-4}	10^{-2}	10^{-4}	10^{-4}	*dilution*
Psychoda phalaenoides	f	x	x	x	x	x		x	
P. phalaenoides	m			x	x			x	
P. zetterstedti	f		x						
P. zetterstedti	m								
P. cinerea	f								
P. sp.	f		x	x			x		
P. trinodulosa	m								
Coproica ferruginata	f								
		x	x	x	x			x	1-Octen-3-ol
		x	x	x	x		x	x	2,6-Dimethylocta-2,6-diene (isomer 1&2)
		x						x	2-Heptanone
				x	x				2-Heptanol
		x		x	x				2-Nonanone
		x	x	x	x		x	x	3,7-Dimethyloct-2-ene (isomer 2)
		x	x	x	x			x	3,7-Dimethyloct-1-ene
		x	x	x	x			x	*α*-Humulene
		x	x	x	x			x	(E)-*β*-Caryophyllene
		x	x	x	x				(+)-*β*-Citronellene
		x	x	x	x	x		x	Indole
		x	x	x	x	x		x	*p*-Cresol
			x	x					6-methyl-5-hepten-2-one
			x						Germacrene D
						x		x	2-Phenylethanol§
						x		x	Dimethyldisulfid§
						x		x	Dimethyltrisulfid§
